# Attenuation of Excess TNF-α Release in Crohn’s Disease by Silencing of iRHOMs 1/2 and the Restoration of TGF-β Mediated Immunosuppression Through Modulation of TACE Trafficking

**DOI:** 10.3389/fimmu.2022.887830

**Published:** 2022-05-02

**Authors:** Taylor J. Louis, Ahmad Qasem, Saleh A. Naser

**Affiliations:** Division of Molecular Microbiology, Burnett School of Biomedical Sciences, College of Medicine, University of Central Florida, Orlando, FL, United States

**Keywords:** TACE (TNF-α converting enzyme), rhomboid proteins, mycobacteria paratuberculosis, Crohn's disease, ectodomain shedding, iRHOM 1/2

## Abstract

TNFα converting enzyme (TACE) is a transmembrane metalloprotease that sheds an assortment of signaling receptors, cytokines, growth factors, and pro-inflammatory mediators. In Crohn’s disease (CD), TACE activity is upregulated, resulting in a marked increase of TNFα secretion and inflammation. Although treatment of CD with TNFα monoclonal antibodies is beneficial, many patients are at risk for acquiring opportunistic infections, and the treatment efficacy of TNFα monoclonal antibodies typically decreases over time. This study investigated an alternative approach for mitigating TNFα release by knocking down TACE membrane translocation in macrophages *via* inhibitory rhomboid proteins 1 and 2 (iRHOMs 1/2) siRNA treatment. First we measured TGFβRII shedding in *ex vivo* plasma samples collected from CD patients and healthy control subjects (N=40 per group). Then, we measured TGFβRII shedding and the expression and production of TGFβ ligand, TNFα, IL-6, IL-1β, IL-10, and total versus membranous TACE *in vitro* with THP-1 derived macrophage infected with *Mycobacterium avium* subspecies *paratuberculosis* (MAP), a highly studied CD-related pathogen. We determined that TGFβRII shedding was significantly higher in CD patients compared to healthy controls [515.52 ± 54.23 pg/mL vs 310.81 ± 43.16 pg/mL, respectively], and MAP-infected CD plasma samples had significantly more TGFβRII shedding (601.83 ± 49.56 pg/mL) than MAP-negative CD samples (430.37 ± 45.73 pg/mL). Moreover, we also determined that TACE production; TGFβ ligand expression and production; and TGFβRII shedding were also higher in MAP-infected THP-1 macrophages. Nevertheless, once we transfected the MAP infected macrophages with iRHOM siRNA, TACE production and membrane localization were significantly decreased, resulting in a significant decrease in TGFβRII shedding; an increase in Smad3 phosphorylation; a decrease in the expression and production of pro-inflammatory cytokines; and a decrease in the expression and production of stricture-associated factor, plasminogen activator inhibitor-1 (PAI-1). Our data clearly demonstrates that the regression of TACE trafficking, *via* iRHOM 1/2 silencing, significantly reduces the release of TNFα and restores the immunosuppressive capabilities of TGFβ signaling, which ultimately reverses inflammatory tissue damage. Accordingly, this study may provide a framework for the creation of newer, safer therapeutic options designed to treat inflammatory autoimmune diseases such as CD and rheumatoid arthritis.

## Introduction

Transforming growth factor beta (TGF-β) is a pleiotropic cytokine that maintains intestinal immune tolerance by suppressing colitis stimulated by flora bacterial antigens (1). Under normal physiological circumstances, non-hematopoietic and immune cells secrete latent TGF-β that is activated by itinerate αvβ integrins within the extracellular space (1). Ensuingly, the activated ligand binds to a heterotetrameric complex embedded in the membranes of T-cells, B-cells, dendritic cells, macrophages, and epithelial cells (1). This interaction elicits a transphosphorylation cascade, which results in the release of anti-inflammatory cytokines and the differentiation of T cells and macrophages into their respective immunosuppressive phenotypes, i.e., T regulatory cells, T helper 17 cell, and M2 macrophages (1–5). Additionally, TGF-β signaling can also elicit the activation of wound repair mechanisms, which regenerates tissue damaged by inflammation (4). However, in spite of these efficacious functions, several studies have indicated that TGF-β signaling is significantly impaired in patients with Crohn’s disease (CD), even though histological analysis of CD intestinal tissues indicate an increased presence of TGF-β ligand (1, 6, 7).

CD is a debilitating inflammatory bowel disease (IBD) characterized by diffuse lesions within the ileum and colon (8). Symptoms of CD include abdominal pain, diarrhea, intestinal wasting, and malnutrition (9). The etiology of CD is historically attributed to environmental triggers and genetic factors (8, 10, 11). However, several groups, including our own, have linked *Mycobacterium avium* spp. *paratuberculosis* (MAP) infection to the development of CD (9, 12, 13).

MAP is a slow growing, obligate intracellular pathogen that enters the body *via* the fecal oral route, and subsequently infects ileal macrophages (12). The resulting pro-inflammatory milieu elicits the recruitment of several effector cells, including cytotoxic T cells, which have the capacity to eliminate itinerate MAP and MAP-infected macrophages (14). Nevertheless, initiation of this cellular response is much slower than the propagation of infection (15). Consequently, MAP bacteria are able to initiate and modulate the resulting immune response (15). A way in which MAP modulates host immunity is by increasing the activity of TNF-α converting enzyme (TACE) (16).

TACE is a transmembrane metalloprotease that sheds an assortment of cytokines, receptors, pro-inflammatory mediators, and growth factors (17). During infection, mannose and toll-like 2 receptors located on the surfaces of macrophages bind to man-nose-capped lipoarabinomannan (ManLAM), a substituent of the mycobacterial cell wall (17–19). This interaction activates the p38 MAPK signaling pathway, which elicits the excessive trafficking of TACE to the cell surface (16, 20–22). However, increased expression of TACE on the membrane surface results in excessive cleavage of membranous TNF-α and TGF-β receptors (16, 23). Therefore, the objective of this study is to find a novel means by which to regress MAP-induced TACE trafficking.

After consulting the literature, we deduced that silencing inhibitory rhomboid proteins 1 and 2 (iRHOMs 1/2) would be an effective way to achieve this goal. iRHOMs 1 and 2 are regulatory co-factors which facilitate TACE trafficking and maturation within the cell (23, 24). By targeting iRHOMs 1/2, instead of directly targeting TACE, we are able to prevent unnecessary off target issues while still mitigating MAP-induced inflammation (25). Additionally, we also decided to investigate the roles of the TGF-β1 ligand isoform and the TGFβRII receptor isoform because TGF-β1 is the predominant isoform secreted by macrophages and is the most important isoform for wound healing, while TGFβRII determines ligand specificity for the receptor complex and initiates the TGF-β signaling cascade (3, 4, 26).

## Materials and Methods

### Clinical Samples

Plasma from peripheral blood samples (4.0 mL K2-EDTA tube) was collected from 100 CD patients (CDAI ≥220 and ≤450) and 40 healthy control subjects and the status of MAP infection was subsequently determined *via* IS900 nPCR as described earlier (27). TGFβRII shedding within the samples was determined by using a TGFBR2 ELISA kit (catalog #EHTGFBR2) per the manufacturer’s instructions (ThermoFisher Scientific, Waltham, MA). Results were assessed using a Multiskan FC plate reader (Fisher Scientific, Waltham, MA) read at absorbance of 450 nm. This study was approved by the University of Central Florida Institutional Review Board #STUDY00003468. All samples were de-identified before handling.

### THP-1 Cell Culture

THP-1 monocytes (ATCC TIB-202) were cultured in ATCC-formulated RPMI-1640 medium supplemented with 0.05 mM 2-mercaptoethanol and 10% fetal bovine serum. Cells were maintained at 37°C in a humidified 5% CO_2_ incubator, when seeded, and allowed to grow in 12-well plates at a density of 4×104 cells per well until confluency was reached. The monocytes were subsequently differentiated into macrophages using phorbol 12-myristate 13-acetate (PMA).

### Bacterial Infection

THP-1 monocytes were plated in a 24-well plate then treated with PMA for 48 hours. Following incubation, the resulting macrophages were infected with 1x107 CFU/mL of MAP and other intracellular pathogens that infect intestinal macrophages, i.e *L. monocytogenes*, *E. coli*, and *K. pneumoniae*, for 24 hours. Cells were subsequently harvested and used for RNA isolation, protein extraction, and phosphorylation activity assessment.

### Quantifying TGF-β Secretion and TGFβRII Shedding

200 uL of cell supernatant was harvested from each *in vitro* experimental group and TGF-β ligand and TGFβRII shedding was determined using an Invitrogen TGF beta-1 Human ELISA Kit (catalog # BMS249-4) and an Invitrogen TGFBR2 Human ELISA Kit (catalog # EHTGFBR2), respectively, per the manufacturer’s instructions (ThermoFisher, Waltham, MA). Results were assessed using a Multiskan FC plate reader (Fisher Scientific, Waltham, MA) read at absorbance of 450 nm. All sets were performed in triplicates.

### Quantifying Total Cellular TACE

24 hours after infection, THP1-macrophages were lysed using a RIPA protein extraction protocol, then 100 uL of the respective cell lysates were added to a microplate well in triplicates. The amount of total cellular TACE was quantified using an Invitrogen Human TACE (ADAM17) ELISA kit (catalog # EHADAM17) per the manufacturer’s instructions (ThermoFisher, Waltham, MA). Results were assessed using a Multiskan FC plate reader (Fisher Scientific, Waltham, MA) read at absorbance of 450 nm. All sets were performed in triplicates.

### Quantifying Transmembrane TACE

24 hours after treatment/infection, we removed the cell media from the THP-1 macrophages and added 5 mL of accutase (ThermoFisher, Waltham, MA) to each respective group. The cells were subsequently incubated for 25 minutes in a humidified, 37°C, 5% CO_2_ incubator, collected, spun down, and all of the accutase was removed. Afterwards, the pellets were washed and transmembrane TACE was isolated using a Mem-PER™ Plus Membrane Protein Extraction Kit (catalog # 89842) per the manufacturer’s instructions (ThermoFisher, Waltham, MA). The amount of TACE within each sample was quantified *via* the Invitrogen Human TACE (ADAM17) ELISA kit (ThermoFisher, Waltham, MA), as mentioned in section 2.5. Results were assessed using a Multiskan FC plate reader (Fisher Scientific, Waltham, MA) read at absorbance of 450 nm. All sets were performed in triplicates.

### RNA Extraction, Reverse Transcription, and Real-Time PCR

Total RNA was extracted and purified from infected/treated THP-1 macrophages using an RNeasy^®^ Mini Kit (Qiagen, Hilden, Germany) per the manufacturer’s instructions, and then the extracted RNA was subjected to a thermal cycler (MyGene Series Peltier) for high-capacity cDNA reverse transcription. 5 μL of the resulting cDNA was mixed with 10 μL of Fast SYBR Green Mastermix (ThermoFisher, Waltham, MA); 1μL of either *TGF-β1*, *TGFβRII*, *TNF-α*, *IL-1β*, *IL-6*, *IL-10*, *NOX-1*, or *Serpine-1* forward and reverse primers, respectively (ThermoFisher, Waltham, MA); and 4 μL of molecular biological grade sterile H2O in a 96-well microamp RT-PCR reaction plate. GAPDH was the control used to obtain baseline CT readings. A 7500 Fast Real-Time PCR System (Applied Biosystems) was used to perform the RT-PCR reaction. Relative mRNA expression levels were calculated using the equation (2^(- ΔΔCT). All sets were performed in triplicates.

### iRHOM siRNA Transfection

First, the iRHOM siRNA powders, sense sequences: RHBDF1 5’-GCGGUAUGGGAAGCUAAAGtt-3’ and RDBDF2 5’- GCUCGAUUGAC-CUGAUCCAtt-3’ (Sigma Aldrich, St. Louis, MO), were suspended in 50 uL of molecular grade sterile H2O, then 3.3 uL of siRNA stock was diluted with 30 uL of Optimem medium (ThermoFisher, Waltham, MA). 9 uL of the resultant iRHOM-Optimem mix was subsequently diluted again with 450 uL Optimem medium. Then, 450 uL of the twice diluted iRHOM siRNA solution was added to a solution containing 27 uL of Lipofectamine^®^ reagent™ (ThermoFisher, Waltham, MA) diluted in 450uL of Optimem medium. 300 uL of the final siRNA transfection mix was added to each treatment well. For the MAP plus iRHOM 1 and 2 wells, 300 uL of iRHOM 1 and 300 uL of iRHOM 2 was added. 24 hours after administering the siRNA transfection mix, we changed the cell media because the Lipofectamine^®^ reagent™ is cytotoxic; infected the cells with 1x107 CFU/mL of MAP, incubated overnight in a humidified, 37°C, 5% CO_2_ incubator; and harvested the cells to latterly use for RNA isolation, protein extraction, and phosphorylation activity measurements. A mock transfection was also performed to investigate the potential effects of the reagents on *TNF-α* and *TACE* expression (please refer to section 3.4 for more details). The difference in gene expression among the three treatment groups was not significant.

Additionally, Silencer™ Negative Control No. 3 siRNA (ThermoFisher, Waltham, MA) was used as a negative control in all trials in the absence and presence of infection or iRHOM siRNA. The difference in TACE production in the negative control siRNA group compared to the other infected and treated groups was insignificant.

### Measuring Phosphorylation Levels of Smad3

Treated/infected THP-1 macrophages were lysed using a RIPA protein extraction protocol, then Smad3 levels within the cell lysates was determined using a SMAD3 (Phospho) (pS423/pS425) Human InstantOne™ ELISA Kit (catalog # 85-86192-11) per the manufacturer’s instruction (ThermoFisher, Waltham, MA). Results were assessed using a Multiskan FC plate reader (Fisher Scientific, Waltham, MA) read at 450 nm. All sets were performed in triplicates.

### Quantifying the Production of Cytokines and PAI-1

Cell supernatant was collected from the infected/treated groups, then allocated into a 96-well plate in triplicates. Each assay (i.e., TNF-α, IL-6, IL-1β, IL-10, and PAI-1 ELISA Assay; ThermoFisher, Waltham, MA) was performed individually according to the manufacturer’s instructions. Results were assessed using a Multiskan FC plate reader (Fisher Scientific, Waltham, MA), read at an absorbance of 450 nm. All sets were performed in triplicates.

### Co-Culturing THP-1 Macrophages With Caco-2 Monolayers

Caco-2 cells (ATCC HTB-37) were seeded in ATCC-formulated Eagle’s minimum essential medium supplemented with 20% fetal bovine serum. THP-1 monocytes were concurrently seeded in ATCC-formulated RPMI-1640 medium supplemented with 0.05 mM 2-mercaptoethanol and 10% fetal bovine serum. Both cell groups were maintained at 37°C in a humidified 5% CO_2_ incubator, respectively. Then the Caco-2 cells were, subsequently cultured in a 12-well plate at a density of 4×104 cells per well for 2 weeks. 4 days prior to the co-culture, THP-1 monocytes were added to well inserts, at a density of 2×52 cells per insert; treated with PMA; and incubated for 48 hours. At this time, the THP-1 monocytes were differentiated into macrophages and, subsequently, transfected with 150 uL of the corresponding iRHOM siRNA mix. The inserts were incubated for an additional 24 hours, then the cell media was changed before 1x107 CFU/mL of MAP was added. Note: cell media was changed prior to infection because the transfection reagent is highly cytotoxic. 24 hours following infection, we added the treated/infected THP-1 inserts to the plated Caco-2 monolayers and incubated the co-culture for an additional 24 hours. Caco-2 cells were subsequently harvested for RNA extraction and oxidative stress studies.

### Quantifying Caco-2 Oxidative Stress

24 hours after the co-culture, we discarded the THP-1 well inserts, and then removed the media from the Caco-2 cells. In its place, we added 5 mL of accutase (ThermoFisher, Waltham, MA) and incubated the plate for 25 minutes in a humidified, 37°C, 5% CO_2_ incubator. After incubation, we collected the solution; spun it down for 5 minutes, 1250 rpm, at room temperature; and removed all of the accutase, leaving only the pellet. RIPA buffer was added directly to the pellets in order to lyse the cells. The amount of NADPH versus NADP within the sample lysates was determined using a NADP^+^/NADPH Assay Kit (catalog # ab65349) per the manufacturer’s instructions (Abcam, Cambridge, UK). Results were assessed using a Multiskan FC plate reader (Fisher Scientific, Waltham, MA), read at an absorbance of 450 nm. All sets were performed in triplicates.

### Visualizing Caco-2 Oxidative Stress

We also performed a DHE fluorescence staining to visualize the oxidative stress experienced by the co-cultured Caco-2 cells as described earlier (28). Briefly, Caco-2 cells and treated/infected THP-1 macrophages were co-cultured in a Falcon™ Chambered Cell Culture Slide (Fisher Scientific, Waltham, MA). After a 24-hour period of incubation, the THP-1 inserts were removed, then the Caco-2 monolayers were washed the with PBS. Afterwards, we fixed the cells to the slides using 4% paraformaldehyde; treated them with 1uM DHE stain (Sigma Aldrich, St. Louis, MO) for 25 min; and then examined them under a Amscope IN480TC-FL-MF603 fluorescent microscope. The average integrated density of red staining, which is indicative of oxidative stress, was measured using the NIH image J program.

### Statistical Analysis

The Kolmogorov–Smirnov normality test was used to test the normal distribution for all values and a two-way analysis of variance (ANOVA) was used to assess significance among experiments, which was followed by a Bonferroni correction test. All experiments were performed in triplicates. *Indicates P-value of less than 0.05.

## Results

### MAP-Positive CD Patients Have Significantly More TGFβRII Shedding in Their Plasma Compared to MAP-Negative CD Patients and Healthy Control Subjects

**Figure 1:**
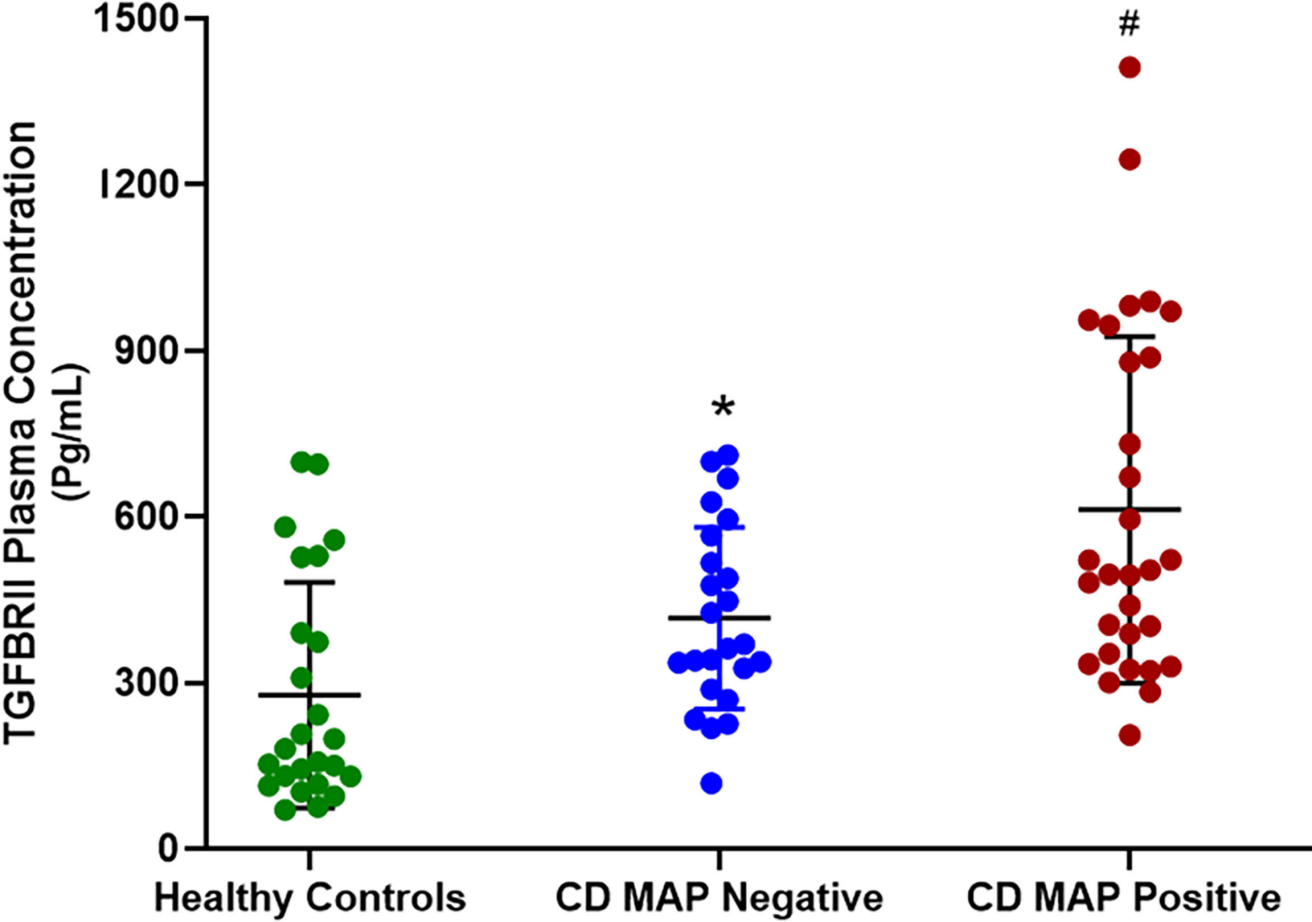
TGFβRII shedding in clinical plasma samples obtained from healthy control subjects, MAP-negative CD patients, and MAP-positive CD patients (*N= 40* per group). The Kolmogorov–Smirnov normality test was used to test the normal distribution for all values and a two-way analysis of variance (ANOVA) was used to assess significance among experiments, which was followed by a Bonferroni correction test. All experiments were performed in triplicates. *P-value < 0.05 compared to healthy controls. ^#^P-value < 0.05 compared to healthy controls and MAP-negative CD patients.

We measured TGFβRII shedding in plasma samples obtained from 40 healthy control subjects, 40 MAP-positive CD patients, and 40 MAP-negative CD patients. Our results indicated that, as a group, the CD patients displayed significantly more TGFβRII shedding compared to the healthy control subjects (515.52 ± 54.23 pg/mL vs 310.81 ± 43.16 pg/mL, respectively). Additionally, within the CD patient population, the MAP-positive patients had even more TGFβRII shedding (601.83 ± 49.56 pg/mL) compared to the MAP-negative CD patients (430.37 ± 45.73 pg/mL) (**Figure 1**).

### MAP Infection Provokes Aberrant TGFβRII Shedding in Macrophages

We measured TGFβRII shedding in THP-1 derived macrophages infected with MAP, *L. monocytogenes*, *E. coli*, and *K. pneumoniae*, respectively, to see if this phenomenon was MAP-specific. All of the aforementioned pathogens are intracellular bacteria that infect intestinal macrophages and were thus deemed appropriate for comparative studies.

**Figure 2:**
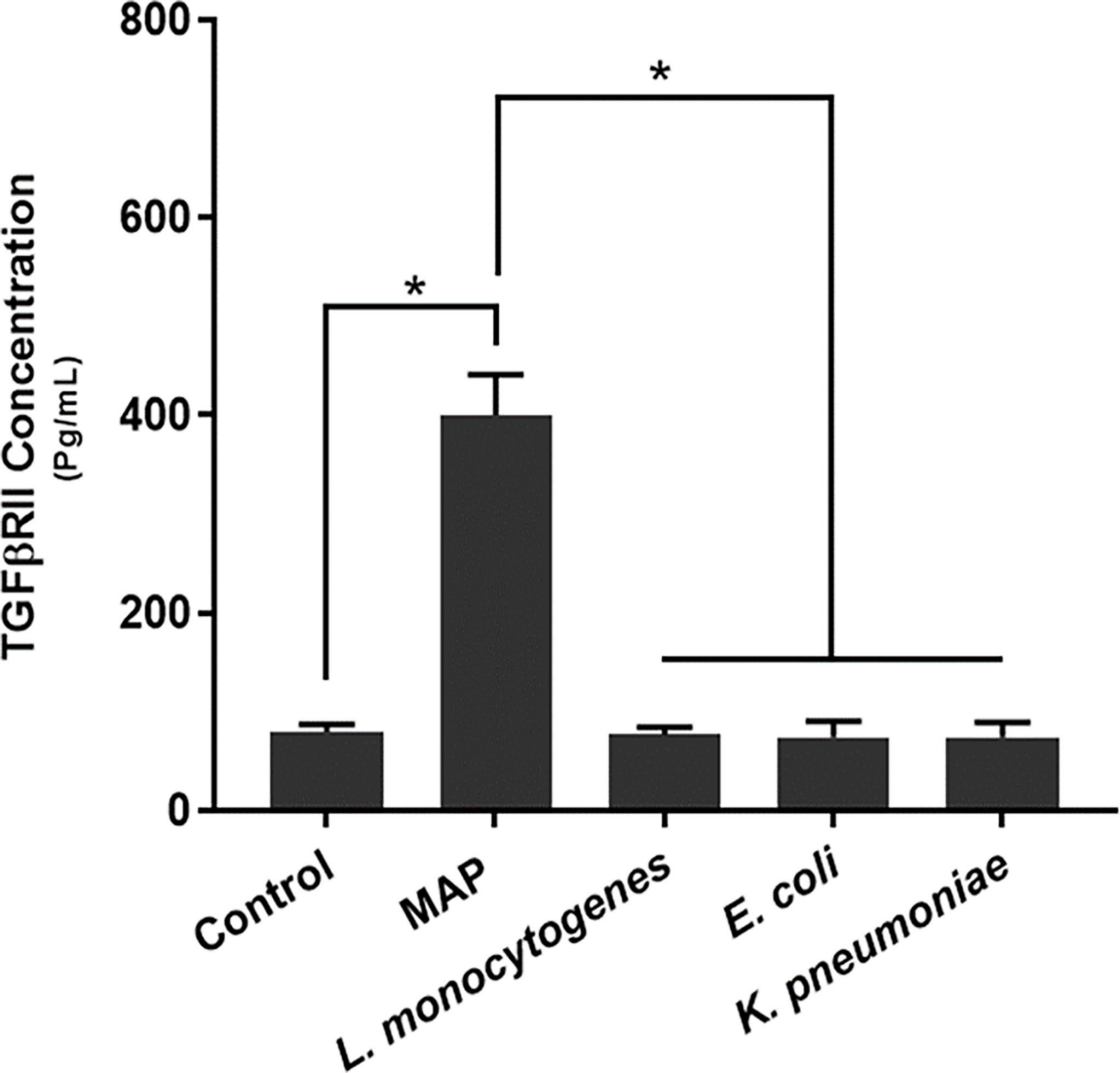
TGFβRII shedding in bacteria infected THP-1 derived macrophages. The Kolmogorov–Smirnov normality test was used to test the normal distribution for all values and a two-way analysis of variance (ANOVA) was used to assess significance among experiments, which was followed by a Bonferroni correction test. All experiments were performed in triplicates. *Indicates P-value of <0.05.

Our results indicated that MAP infection elicits significantly more TGFβRII shedding (400.36 ± 40.85 pg/mL) than the control group (79.86 ± 7.61 pg/mL) and THP-1 macrophages infected with *L. monocytogenes* (76.85 ± 7.42 pg/mL), *E. coli* (74.14 ± 16.49 pg/mL), and *K. pneumoniae* (74.43 ± 14.8 pg/mL) (**Figure 2**).

### MAP Infection Elicits the Expression and Production of TGF-β Ligand by Macrophages

**Figure 3:**
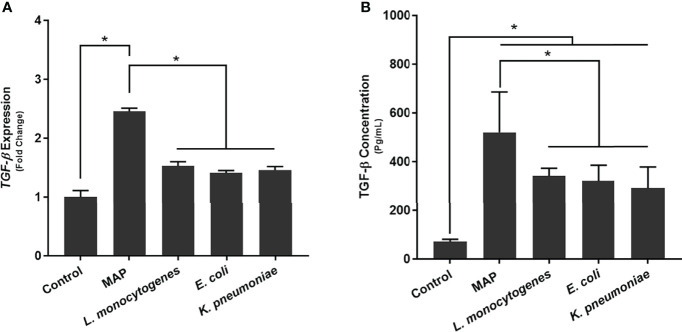
The **(A)** expression and **(B)** production of TGF-β ligand in bacteria infected THP-1 derived macrophages. The Kolmogorov–Smirnov normality test was used to test the normal distribution for all values and a two-way analysis of variance (ANOVA) was used to assess significance among experiments, which was followed by a Bonferroni correction test. All experiments were performed in triplicates. *Indicates P-value of less than 0.05.

To determine how MAP infection affects the expression and production of TGF-β ligand compared to other pathogens, we infected THP-1 derived macrophages with MAP, *L. monocytogenes*, *E. coli*, and *K. pneumoniae*, respectively. Our results indicated that, in addition to eliciting aberrant TGFβRII shedding, MAP infection also elicits the exorbitant expression and production of TGF-β ligand (2.46 fold, 520.16 ± 166.08 pg/mL); which was significantly more than the expression and production values obtained from the control group (1 fold, 70.46 ± 10.64 pg/mL) and the other infected groups: *L. monocytogenes* (1.53 fold, 341.09 ± 31.68 pg/mL), *E. coli* (1.41 fold, 320.95 ± 64.74 pg/mL), and *K. pneumoniae* (1.46 fold, 291.15 ± 87.07 pg/mL) (**Figure 3**).

### Validation of the iRHOM 1/2 siRNA Transfection

**Figure 4:**
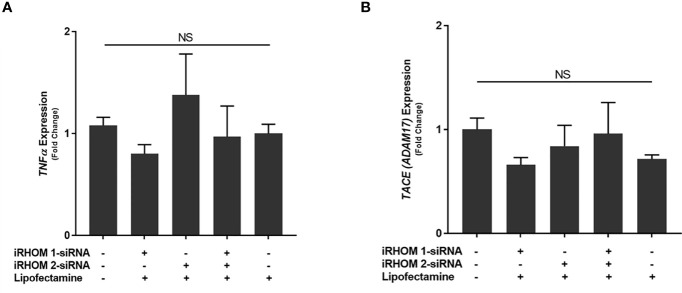
The impact of an iRHOM 1/2 siRNA mock transfection of the expression of **(A)** *TNF-α* and **(B)** *TACE*. The Kolmogorov–Smirnov normality test was used to test the normal distribution for all values and a two-way analysis of variance (ANOVA) was used to assess significance among experiments, which was followed by a Bonferroni correction test. All experiments were performed in triplicates. The difference in gene expression among the control and treated groups was not significant (NS).

To determine if the iRHOM siRNA transfection reagents affected *TNF-α* and *TACE* expression in non-infected THP-1 macrophages, we performed a mock transfection in which we subjected the cell cultures to the transfection reagent (Lipofectamine^®^ reagent™) and iRHOM siRNA suspended in the transfection reagent (**Figure 4**). Similar to the mock transfection, transfection with a negative control siRNA showed no changes in TACE production. From this study, we confirmed that the transfection reagents have no effect on the normal expression of *TNF-α* (**Figure 4** **A**) and *TACE* (**Figure 4** **B**) within THP-1 macrophages and is, therefore, considered inert in the absence of MAP infection.

### Silencing the Expression of iRHOMs 1 and 2 in MAP-Infected Macrophages Regresses TACE Trafficking and TGFβRII Shedding

**Figure 5:**
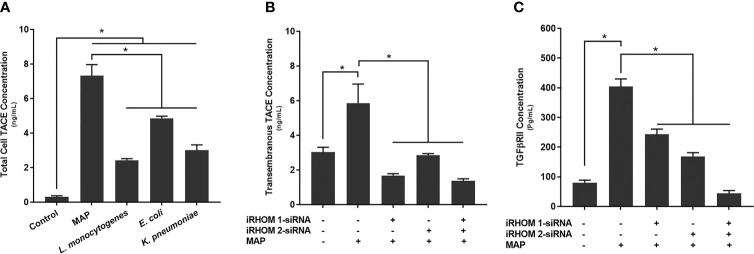
The total concentration of TACE within bacteria infected cells **(A)** and the impact of TACE regression on the concentration of **(B)** transmembrane TACE levels and **(C)** TGFβRII shedding. The Kolmogorov–Smirnov normality test was used to test the normal distribution for all values and a two-way analysis of variance (ANOVA) was used to assess significance among experiments, which was followed by a Bonferroni correction test. All experiments were performed in triplicates. *Indicates P-value of less than 0.05.

To determine how MAP infection potentially elicits the attenuation of TGF-β signaling, we first infected THP-1 macrophages with MAP, *L. monocytogenes*, *E. coli*, and *K. pneumoniae*, respectively, then we quantified the presence of TACE within the whole cell lysates. From this study, we were able to ascertain that MAP infection increases the global presence of TACE within THP-1 macrophages (7.32 ± 0.65 ng/mL) significantly more than the control group (0.3 ± 0.07 ng/mL) and cells infected *L. monocytogenes* (2.43 ± 0.09 ng/mL), *E. coli* (4.86 ± 0.12 ng/mL), and *K. pneumoniae* (3 ± 0.32 ng/mL) (**Figure 5** **A**).

In a similar study, we also observed an elevation in TACE membrane trafficking in MAP-infected THP-1 macrophages (5.86 ± 1.10 ng/mL) compared to the control group (3.03 ± 0.28 ng/mL) (**Figure 5** **B**), resulting in excessive TGFβRII shedding (control: 79.87 ± 8.90 pg/mL, MAP: 404 ± 25.7 pg/mL) (**Figure 5** **C**). Nevertheless, when we experimentally regressed MAP-induced TACE trafficking, *via* the iRHOM siRNA treatments (iRHOM 1: 1.68 ± 0.11 ng/mL, iRHOM 2: 2.85 ± 0.10 ng/mL, iRHOM 1/2: 1.37 ± 0.12 ng/mL) (**Figure 5** **B**), we observed a significant decrease in TGFβRII shedding (iRHOM 1: 242.87 ± 17.60 pg/mL, iRHOM 2: 168.88 ± 12.40 pg/mL, iRHOM 1/2: 44.23 ± 9.40 pg/mL) (**Figure 5** **C**).

### Regression of TACE Trafficking Restores Smad3 Phosphorylation in MAP-Infected Macrophages

**Figure 6:**
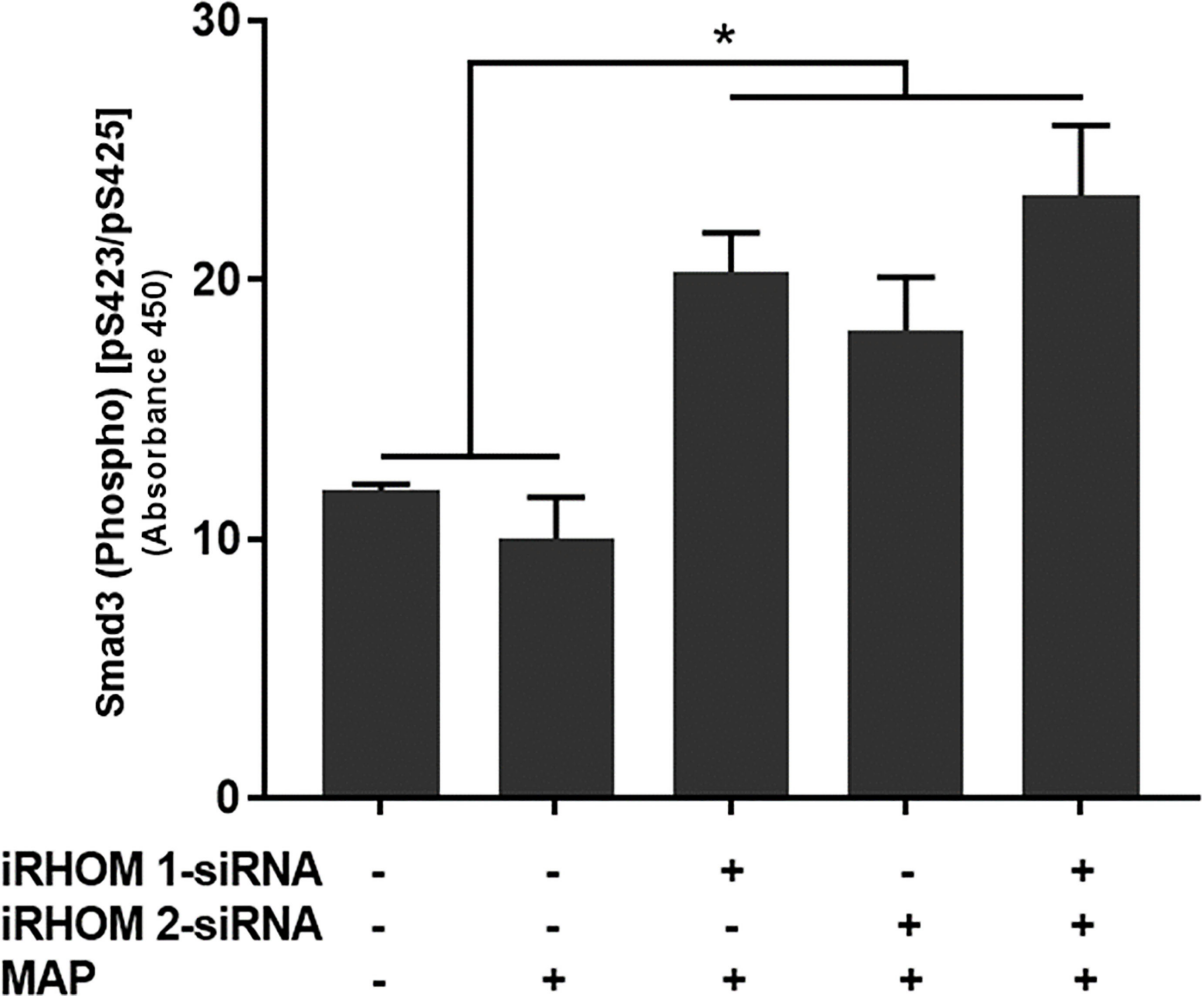
The impact of TACE regression on the downstream phosphorylation of Smad3. The Kolmogorov–Smirnov normality test was used to test the normal distribution for all values and a two-way analysis of variance (ANOVA) was used to assess significance among experiments, which was followed by a Bonferroni correction test. All experiments were performed in triplicates. *Indicates P-value of less than 0.05.

To determine if the siRNA treatments also rescued downstream signaling within the TGF-β pathway, we assessed the intracellular phosphorylation of Smad3 in treat/infected THP-1 macrophages. Based on our results, the iRHOM siRNA treatments increased the incidence of downstream Smad3 phosphorylation significantly more than the non-treated control and MAP-only groups (control: 11.85 ± 0.27, MAP: 10 ± 1.60, iRHOM 1: 20.31 ± 1.50, iRHOM 2: 18.04 ± 2.05, iRHOM 1/2: 23.25 ± 2.70) (**Figure 6**).

### Regression of TACE Trafficking Significantly Reduces the Pro-Inflammatory Milieu Elicited by MAP-Infected Macrophages

**Figure 7:**
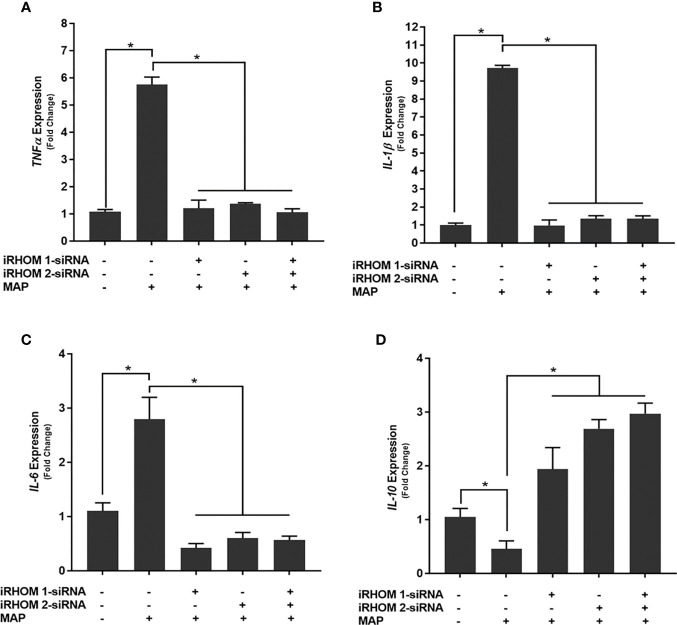
The impact of TACE regression on the expression of **(A)** TNFα, **(B)** IL-1β, **(C)** IL-6, and **(D)** IL-10. The Kolmogorov–Smirnov normality test was used to test the normal distribution for all values and a two-way analysis of variance (ANOVA) was used to assess significance among experiments, which was followed by a Bonferroni correction test. All experiments were performed in triplicates. *Indicates P-value of less than 0.05.

To determine if siRNA-mediated restoration of TGF-β signaling suppresses the pro-inflammatory milieu, we quantified the expression and production of select pro- and anti-inflammatory cytokines after treating/infecting the THP-1 macrophages with iRHOM siRNA and MAP, respectively. Our results indicated that the siRNA treatments effectively restored TGF-β mediated immunosuppression as evidenced by the decrease in pro-inflammatory cytokine (i.e., TNF-α, IL-1β, and IL-6) expression and production (**Figures 7** **A–C** and **Table 1**), concurrent to the increased expression and production of the anti-inflammatory cytokine, IL-10 (**Figure 7** **D** and **Table 1**).

**Table 1:** The impact of TACE regression on the production of TNF-α, IL-1β, IL-6, and IL-10.

Infection and treatment	TNF-α ± SD (pg/mL)	IL-1β ± SD (pg/mL)	IL-6 ± SD (pg/mL)	IL-10 ± SD (pg/mL)
Control (no infection)	33.47 ± 1.71	16.1 ± 1.47	1378.90 ± 11.0
MAP (1x10^7^ CFU/mL)	1683.40 ± 71.50	1040.00 ± 234.00	1505.00 ± 95.30	163.60 ± 4.70
iRHOM 1 siRNA + MAP	90.20 ± 9.32*	47.70 ± 15.09*	43.45 ± 10.20*	1868.70 ± 58.20*
iRHOM 2 siRNA + MAP	68.60 ± 0.53*	68.48 ± 10.90*	40.10 ± 17.10*	1457.40 ± 45.90*
iRHOM 1 siRNA + iRHOM 2 siRNA + MAP	86.00 ± 5.10*	69.74 ± 15.98*	55.58 ± 15.50*	1366.90 ± 131.10*

*Indicates P-value of less than 0.05.

### Regression of TACE Trafficking Significantly Reduces Oxidative Stress Experienced by Intestinal Epithelial Cells Co-Cultured With MAP-Infected Macrophages

**Figure 8:**
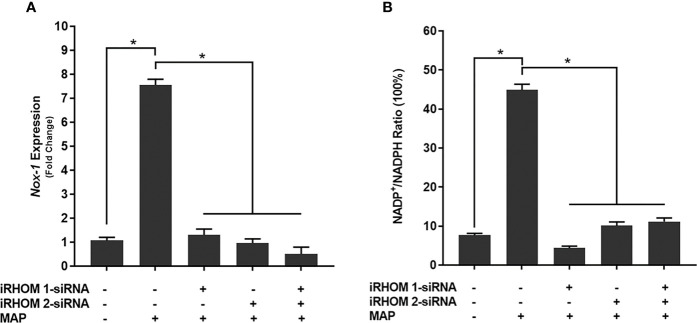
How regressing TACE trafficking affects **(A)** the expression of NADPH oxidase-1 (*NOX-1*) and **(B)** the resulting ratio of NADP^+^/NADPH in co-cultured Caco-2 monolayers. The Kolmogorov–Smirnov normality test was used to test the normal distribution for all values and a two-way analysis of variance (ANOVA) was used to assess significance among experiments, which was followed by a Bonferroni correction test. All experiments were performed in triplicates. *Indicates P-value of less than 0.05.

To determine if the iRHOM siRNA treatments mitigated Caco-2 oxidative stress, we co-cultured siRNA-treated/MAP-infected THP-1 macrophages with Caco-2 monolayers and, subsequently, assessed the expression of NADPH oxidase-1 (*NOX-1*) (**Figure 8** **A**) and the resulting ratio of oxidized versus non-oxidized NADPH within the Caco-2 monolayers (**Figure 8** **B** and **Table 2**). Based on our results, the iRHOM siRNA treatments significantly reduced the expression of *NOX-1* [control: 1.07-fold, MAP: 7.56-fold, iRHOM 1: 1.31-fold, iRHOM 2: 0.97-fold, iRHOM 1/2: 0.51-fold] and the resulting oxidation of NADPH in the Caco-2 monolayers [control: 7.68 ± 0.46, MAP: 44.93 ± 1.41, iRHOM 1: 4.38 ± 0.48, iRHOM 2: 10.10 ± 0.96, iRHOM 1/2: 11.16 ± 0.93], indicating that our treatment effectively reduced oxidative stress.

**Table 2:** The impact of TACE regression on the oxidation of NADPH.

Infection and treatment	NADPH/(Total NADP + NADPH) Ratio	SD
Control (no infection)	92.86	19.58
MAP (1x10^7^ CFU/mL)	69.00	6.89
iRHOM 1 siRNA + MAP	95.80*	9.44
iRHOM 2 siRNA + MAP	90.76*	7.85
iRHOM 1 siRNA + iRHOM 2 siRNA + MAP	89.96*	7.76

*Indicates P-value of less than 0.05.

**Figure 9:**
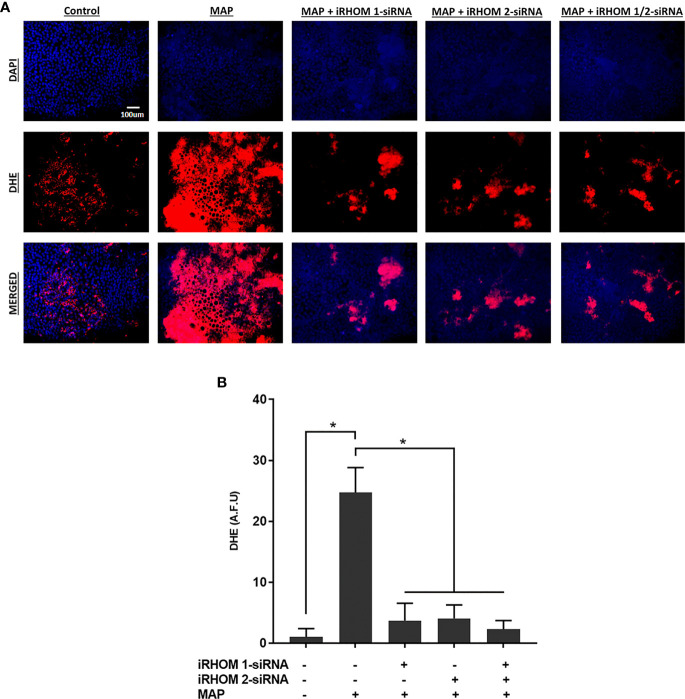
The impact of TACE regression on oxidative stress experienced by co-cultured Caco-2 monolayers. **(A)** The total nuclei are stained with DAPI blue and the DHE positive cells are stained in red; the merged cells are presented in pink. **(B)** The quantitative corrected DHE fluorescence integrated density from Caco-2 cells co-cultured with control and treated THP-1 macrophages. The Kolmogorov–Smirnov normality test was used to test the normal distribution for all values and a two-way analysis of variance (ANOVA) was used to assess significance among experiments, which was followed by a Bonferroni correction test. All experiments were performed in triplicates. *Indicates P-value of less than 0.05. All DHE fluorescence staining experiments were performed in triplicates.

Moreover, we stained co-cultured Caco-2 cells with dihydroethidium (DHE) to visualize the resulting oxidative stress (**Figure 9** **A**) and obtained similar results: the control cells had a fluorescence value of 1.00 ± 1.39, the MAP cells had a fluorescence value of 24.75 ± 4.07, the iRHOM 1 group had a fluorescence value of 3.72 ± 2.82, the iRHOM 2 group had a fluorescence value of 4.07 ± 2.19, and the iRHOM 1/2 group had a fluorescence value of 2.30 ± 1.40 (**Figure 9** **B**).

### Regression of TACE Trafficking Significantly Reduces the Expression and Production of a Stricture-Associated Protein, PAI-1, by MAP-Infected Macrophages

**Figure 10:**
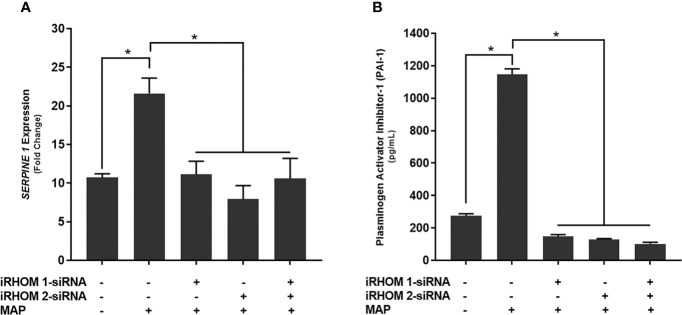
The impact of TACE regression on the **(A)** expression and **(B)** production of plasminogen activator inhibitor-1 (PAI-1). The Kolmogorov–Smirnov normality test was used to test the normal distribution for all values and a two-way analysis of variance (ANOVA) was used to assess significance among experiments, which was followed by a Bonferroni correction test. All experiments were performed in triplicates. *Indicates P-value of less than 0.05.

Finally, we wanted to determine if treating MAP-infected THP-1 macrophages with iRHOM siRNA could reduce the expression and production of plasminogen activator inhibitor-1 (PAI-1); a pro-fibrotic protein known to cause aberrant stricture formation in CD patients (29). Based on our results, the iRHOM siRNA treatments significantly reduced the expression (**Figure 10** **A**) and production (**Figure 10** **B**) of PAI-1 [iRHOM 1: 11.12-fold, 147.7 ± 11.2; iRHOM 2: 7.93-fold, 128.4 ± 5.6; iRHOM 1/2: 10.60-fold, 101.5 ± 10.6] compared to the non-treated groups [control: 10.72-fold, 275 ± 12.2; MAP: 21.60-fold, 1147 ± 34].

## Discussion

Inflammatory bowel diseases are a group of debilitating autoimmune disorders that affect roughly 6 to 8 million people around the globe (30). The annual incidence of these conditions continues to rise at a staggering rate, resulting in substantial human and economic costs (31). The most popular therapies for the treatment of inflammatory bowel diseases are TNF-α monoclonal antibodies (32). These biologics effectively relieve intestinal inflammation by neutralizing membranous and secreted TNF-α (33). However, in the case of MAP-associated CD, this can be especially deleterious.

An estimated 46% of CD patients are infected with MAP (34). This means that a large subpopulation of CD patients may not benefit from the long-term use of TNF-α monoclonal antibodies because it impedes the host’s ability to effectively mitigate infection, which is, ironically, the causative agent for disease (34–36). Accordingly, MAP-infected CD patients receiving TNF-α monoclonal antibodies tend to experience longer, more onerous periods of disease relapse with an increased likelihood of stricture formation (9). In consideration of the abovementioned, our group set out to investigate an alternative approach to regress TNF-α affiliated inflammation in interim of MAP-associated CD.

**Figure 11:**
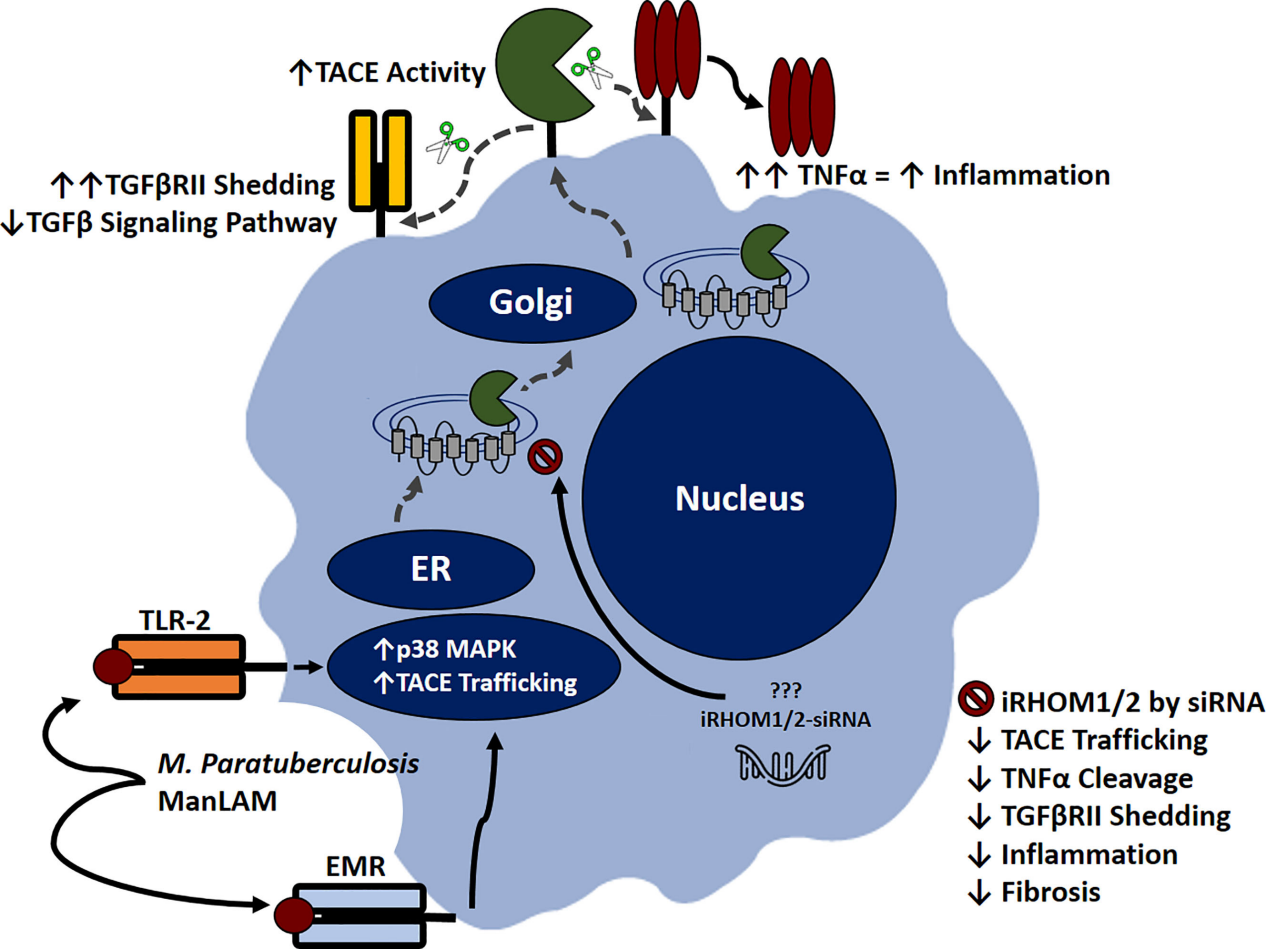
Inhibitory rhomboid proteins (iRHOM1/2) regulate TNF-α converting enzyme (TACE) membrane trafficking. Macrophages recognize *M. paratuberculosis* ManLAM through toll-like 2 receptors (TLR-2) and external mannose receptors (EMR). Upon binding, the p38 MAPK pathway is activated, which facilitates TACE trafficking from the ER to the cell surface *via* iRHOM1/2 regulation. Increasing TACE activity cleaves transmembranous TNF-α and induces TGFβRII shedding, which exacerbates inflammation and attenuates TGF-β signaling pathway. Silencing iRHOM1/2 downregulates TACE trafficking and maturation, which consequently decreases TNF-α cleavage and restores TGF-β signaling pathway.

Based on our results, we were able to ascertain that MAP-infection elicits increased TACE production, membrane trafficking, and protease activity in macrophages, resulting in exorbitant TNF-α release and TGFβRII shedding. The subsequent accumulation of TNF-α within the extracellular space will recruit and activate other effector cells; thereby exacerbating the pro-inflammatory milieu (**Figure 11**) (14).

Additionally, the attenuation of TGF-β signaling prevents the resolution of inflammation, resulting in Caco-2 oxidative stress and improper wound healing. Collectively, these occurrences contribute to the normal pathogenesis of MAP-affiliated CD. Nevertheless, the utilization of iRHOM siRNA treatments in interim of infection significantly regresses TACE trafficking, TNF-α release, and TGFβRII shedding. The significance of this is substantial, since moderated TNF-α release restores the host’s ability to form granulomas, resulting in effective MAP containment (34). Moreover, the decreased presence of circulating MAP will trigger TGF-β mediated immunosuppression, which in turn, resolves MAP-induced colitis and makes allowances for proper wound healing (4).

For many years, researchers have known about the therapeutic potential of interfering RNAs, however, significant obstacles in pharmacokinetics prevented FDA approval (37). Artificially prepared siRNAs are large, anionic molecules with a low bioavailability, a small half-life, and inept target site localization (37). As a result, researchers deduced two way to optimize siRNA drug delivery: chemical modification or nanocarrier utilization (37). Chemical modifications increase the molecule’s hydrophobicity and provides protection against nucleases and phosphodiesterases (37). Likewise, nanocarriers, such as liposomes, polymeric nanoparticles, dendrimers, micelles, metallic nanoparticles, niosomes, oligonucleotide nanoparticles, and albumin-based nanoparticles, increase siRNA delivery by improving the drug’s ability to permeate the cell (37). At the time of drafting this manuscript, 7 siRNA-based drugs were approved for the treatment of rare or orphaned diseases; and 3 other siRNA-based drugs were undergoing late-stage clinical trials (37). The most popular modification of the abovementioned drugs was ligand bio-conjugation (37, 38).

Ligand conjugation is a very effective modification because the attached ligand can bind specifically to receptors associated with the target cells (38). Once this interaction occurs, the ligand-siRNA conjugate is rapidly endocytosed, and the ligand is disassociated from the siRNA *via* endosome acidification (38). This cytosol-specific delivery of siRNA drugs decreases toxicity, immunogenicity, and does not require the concomitant use of anti-inflammatory drugs, like the lipid-based nanocarrier siRNA drugs necessitate (37). Moreover, bio-conjugated siRNAs have increased bioavailability, decreased off-target effects, and offer more convenient dosing options (i.e., self- administrable subcutaneous and topical products) (37). These advantages are not possible with novel therapeutic classes, such as monoclonal antibodies (37).

In the future, we plan to test the efficacy of the iRHOM siRNA treatments *in vivo* and optimize drug delivery. Several studies have indicated that iron scavenging receptors, such as SCARA5, are upregulated on the surfaces of MAP-infected macrophages (39–41). Therefore, if we conjugate ferritin to the iRHOM siRNAs, this should effectively deliver the treatment to the cytosol of the effected cells (40). Collectively, the concomitant use of iRHOM siRNAs with an anti-MAP triple antibiotic should mitigate inflammation and sterilize MAP encapsulated granulomas, thereby eradicating MAP-induced CD (34, 35, 42).

## Data Availability Statement

The raw data supporting the conclusions of this article will be made available by the authors, without undue reservation.

## Ethics Statement

The studies involving human participants were reviewed and approved by University of Central Florida Institutional Review Board (STUDY00003468). The patients/participants provided their written informed consent to participate in this study.

## Author Contributions

Conceptualization, TL, AQ, and SN. Formal analysis, TL and AQ. Funding acquisition, SN. Methodology, TL, AQ, and SN. Supervision, SN. Writing - original draft, TL. Writing - review and editing, AQ and SN. All authors have read and agreed to the submitted version of the manuscript.

## Funding

This study was supported in part by the Florida legislative grant.

## Conflict of Interest

The authors declare that the research was conducted in the absence of any commercial or financial relationships that could be construed as a potential conflict of interest.

## Publisher’s Note

All claims expressed in this article are solely those of the authors and do not necessarily represent those of their affiliated organizations, or those of the publisher, the editors and the reviewers. Any product that may be evaluated in this article, or claim that may be made by its manufacturer, is not guaranteed or endorsed by the publisher.

## Abbreviations

CD, Crohn’s disease; EMR, External mannose receptor, FDA, United States Food and Drug Administration; IBD, Inflammatory bowel disease; iRHOMs 1/2, Inhibitory rhomboid proteins 1 and 2; MAP, Mycobacterium avium subspecies paratuberculosis; NOX-1, NADPH oxidase-1; PAI-1, Plasminogen activator inhibitor-1; PMA, Phorbol 12-myristate 13-acetate; RHB-104, anti-MAP triple antibiotic; siRNA, Small interfering RNA; TACE, TNF-α converting enzyme; TGF-β, Transforming growth factor beta; TGFβRII, Transforming growth factor beta receptor II; TNF-α, Tumor necrosis factor alpha; TLR-2, Toll like receptor-2.
